# Novelties and Improvements in Medicine

**Published:** 1867-09

**Authors:** Henry Gibbons


*Novelties and Improvements in Medicine.* By HENRY GIBBONS, M. D.

THE LARYNGOSCOPE.

The speculum, in its various forms, brings into view those objects alone which are in a direct line with the eye. As the larynx is not in a direct line, it can be examined only by an apparatus which enables us, as some one has expressed it, to "see round a corner." Such is the Laryngoscope. It does not, however, bring the larynx itself into view, but the image of the larynx, reflected from a mirror. The little mirror of the dentist, with which he explores the back parts of the teeth, and which was used in the days of Celsus, is the basis of the Laryngoscope.

More than fifty years ago, Bozzini, a German physician, invented what he called a *Light-Conductor,* or an "Apparatus for the Illumination of the Internal Cavities and Spaces in the Living Animal Body." The idea was caught up with avidity by some medical inquirers, and carried to the absurd extreme of supposing that not merely the outlets of the body, but even the internal viscera could be inspected by the apparatus. The Faculty of Physicians of Vienna condemned the invention, and it passed away.

The subject was revived at different times. In 1829, Dr. Babington exhibited at the Hunterian Society of London an instrument which he called a *Glottiscope*, being a near approach to the present Laryngoscope. A similar instrument was invented in Paris, by means of which it was claimed that the vocal cords could be seen. But as late as 1837, Trousseau devoted several pages to prove that this was impossible, and that the epiglottis formed an insuperable impediment to a view of the interior of the larynx.

In 1854, Garcia, a singing-master in London, conceived the novel idea of observing in his own person the movements of the interior of the larnyx during singing. Garcia appears to have been claimed by as many homes as the blind poet of Greece. He resided in London, was a Frenchman by birth, and a Spaniard by descent. His investigations were made wholly on himself, and he succeeded fully in observing and describing the action of the vocal cords during inspiration and vocalization. He employed two mirrors, a small one fixed to a long stem suitably bent, and applied to the pharynx so as to receive the image of the larynx from beneath, and a large one which served the double purpose of throwing the light on the little mirror and enabling him to see the image formed on it. This was substantially the apparatus of Babington.

It was reserved for professor Czermak, of Pesth, in 1857, to take advantage of the inventions and observations of his predecessors, and to improve the instrument, and establish its character as a means of diagnosis and treatment. It has been hinted that he owed his success and the immortality which it has secured for hin, to his own remarkably capacious pharynx, small tonsils and uvula, and large laryngeal aperture, which enabled him to practice with facility on his own person. Although many ingenions persons in all parts of the world have been constantly engaged on the subject, they have made no improvement on the apparatus of Czermak.

The mirror which is designed to receive and reflect the image of the larynx is round or oval in form, and something less than an inch in diameter. It is attached to a handle several inches in length, at an obtuse angle corresponding with the position of the roof of the pharynx in relation to the horizontal palate. The back of the mirror is pressed up against the uvula and into the cavity of the pharynx, from which position it looks down upon the glottis, and receives the image of the laryngeal aperture and its surroundings. In some instances, but not often, it is necessary to use a spatula to depress the tongue.

Mirrors of various forms and sizes are employed to meet particular cases. They may be made of metal or of looking glass, the latter being preferred. Before introduction, the mirror is warmed, to prevent the condensation of moisture on its surface.

A second mirror is so applied as to direct a volume of light upon the little mirror resting in the pharynx, and so upon the parts to be inspected. A circular mirror three or four inches in diameter is employed for the purpose, having in its centre a small hole through which the observer looks, with one eye, and sees how to illuminate the small mirror, and to take note of what it reveals. The large illuminating mirror is fastened on the forehead of the observer by a sort of spectacle frame, so that the hole in its centre shall correspond with one of his eyes. If the sun-light be used, a plane surface is proper, but if artificial light, a concave one. The artificial light, of whatever kind, stands at one side of the patient's head. Lanterns and contrivances of various kinds, less simple than that described, have been invented, to suit the fancy of observers.

It will scarcely be expected that the examination of the larynx as described can be readily accomplished without some tact and experience. There are several important matters to be observed in the process which it is not necessary to introduce here, as my only purpose is to give the reader a general idea of the subject. Any one undertaking the performance would equip himself by minute study of some treatise on laryngoscopy.*

"In some cases, on introducing the laryngeal mirror, only the epiglottis may be visible; while in others the entire length of the vocal cords, the ventricular bands (false vocal cords,) the small cartilage above the glottis, the large cricoid cartilage, the rings of the trachea, and perhaps even the bifurcation of the bronci below it, can be seen with perfect distinctness. The view varies in different cases between these two extremes."

From what has been stated, it is easy to form an estimate of the value of this means of diagnosis, and to appreciate, also, the value of the apparatus as an aid to treatment. Thus, applications are made to the larynx with skill and accuracy, in the form of solutions, powders, nitrate of silver and other escharotics, and galvanism; the parts are scarified, and morbid growths are extirpated. The reader will excuse me if I introduce a few illustrations of the mode of treatment, taken from the works referred to.

Case 1. *Cystic Tumor cured by puncture.*—The patient was nearly strangled by some obstruction in the larynx, which, on examination, proved to be a large, soft tumor, concealing the glottis. The tumor was opened by a sharp-pointed bistory, partially surrounded with sticking-plaster. A sudden gush of glairy fluid took place, with a little pus and blood. All the symptoms were at once relieved, and in the evening he was singing in bed. In a few days he was perfectly well, and so continued. (Durham.)

Case 2. *Chronic Edema; Scarification.*—C. C., aged 22, had great dyspnea, hoarseness and pain in the throat for more than two years. For more than a year had not been able to lie down at night. A large tumor was seen projecting across the

---

* For this purpose, see "The Laryngoscope in diseases of the throat," by Mackenzie, or "Rhinoscopy and Laryngoscopy" by Semeleder.

larynx, of a deep red color. Diagnosis, chronic edema of larynx. A strong solution of nitrate of silver was applied every other day for a month without much benefit. Then the tumor was scarified, which caused the discharge of a considerable quantity of blood and frothy fluid. Two days afterwards, no material change having occurred, the tumor was again freely lanced. Next day he was greatly relieved; had slept well for some hours and awoke refreshed and comfortable—a pleasure he had not known for more than two years. Swelling much diminished. Scarification repeated. In one week from the first scarification, he felt quite well, and wished to go to work. The voice was a little horse at first, but soon became natural, and the respiration entirely free from embarrassment. (Mackenzie.)

Case 3. *Warty Growths removed by Forceps.*—Mrs. A., aged 35, had not been able to speak above a whisper for two years. On examination, a number of warty excrescences were seen on and beneath the vocal cords. A strong solution of nitrate of silver produced too much dyspnea to warrant its continued use. The forceps were resorted to, and after repeated trials and much difficulty the excrescences were entirely removed. A month afterwards the voice was completely restored, and she continued well. (Mackenzie.)

Other cases are on record still more striking, the voice being speedily restored by the removal of warts or polypi after five, seven, or nine years of complete aphonia. In one instance, a child was said to have swallowed a fish-bone, and not being relieved by the probang and other means, and croupy symptoms intervening, the laryngoscope disclosed a small bone nearly concealed behind the larynx. It was removed, though not without much difficulty.

Important negative results are sometimes obtained by the use of the laryngoscope. In a number of instances in which trachcotomy was contemplated for the relief of dyspnea, the instrument revealed no obstruction in the throat, and consequently led to the abandonment of the operation. Aortic aneurism in some of these cases was finally diagnosticated.

Galvanism, applied to the vocal cords, has been followed by some remarkable results, in functional aphonia and in vocal weakness without structural disease. An instrument was introduced by Mackenzie, and has been extensively employed by himself and others, by means of which the electric current can be applied to the arytenoid cartilages so as to stimulate both branches of the pneumogastric nerve. One of Mackenzie's patients was a lady who had lost her voice from a cold, and had not spoken above a whisper for two years. The vocal cords were found to be perfectly healthy though relaxed. A single application of electricity restored the voice immediately. In another patient, whose loss of voice was of two years' standing, the first application was unsuccessful; but after the second, an improvement began, which resulted in complete recovery of voice in about a month.

The cases that I have cited are not to be taken as an impartial exhibit of the results of laryngoscopy, but rather as illustrations of what can be done under favorable circumstances. Many times relief has been attained only by months of laborious and persevering treatment, and it is scarcely necessary to add that many cases resist all treatment. It is clear, however, that as a means of diagnosis, the instrument is a complete success. A similar apparatus has been applied to the examination of the esophagus. By holding the orifice open with forceps, Semeleder succeeded, after a few attempts, in seeing as far down as the lowest edge of the cricoid cartilage, or more than an inch. He is confident that he could explore it still farther. For the satisfaction of persons who are fond of "sweet sounds," it may be well to mention the term ÆSOPHAGOSCOPY in this connection.

RHINOSCOPY refers to a similar exploration of the posterior nares and the orifices of the eustachian tubes. For this purpose the mirror must be smaller and fixed at a right angle with the handle. It is illuminated in the same way as before. A spatula for depressing the tongue is required, and sometimes a hook for drawing forward the uvula. In affections of the nares, such as polypus and ozena, and in obstructions of the eustachian tube, this means of exploration is useful for diagnosis and also for treatment. To Czermak and Semeleder belongs the credit of establishing rhinoscopy as a special department. Mackenzie describes a case of ozena of two years' standing, caused by ulcers on the vomer and right middle turbinated bone, which was cured by local treatment applied by the aid of the mirror.

We should not be surprised if rhinoscopy were to come into general use sooner than laryngoscopy. It is easier of application; the parts involved are more accessible; and their diseases are more simple, requiring less skill in surgical manipulation. Cases of ozena causing great annoyance and trouble to patients as well as to physicians, are always on hand. A more successful plan of treatment than formerly existed would be a great blessing to both parties.—*Pacific Med. Journal.*

